# Relationship and prognostic importance of thyroid hormone and N-terminal pro-B-Type natriuretic peptide for patients after acute coronary syndromes: a longitudinal observational study

**DOI:** 10.1186/s12872-016-0226-2

**Published:** 2016-02-18

**Authors:** Julija Brozaitiene, Narseta Mickuviene, Aurelija Podlipskyte, Julius Burkauskas, Robertas Bunevicius

**Affiliations:** Behavioral Medicine Institute, Lithuanian University of Health Sciences, Palanga, Lithuania

**Keywords:** NT-pro-BNP, Thyroid hormones, Mortality, Coronary artery disease

## Abstract

**Background:**

Altered thyroid function and increased rates of N-terminal pro-B-Type natriuretic peptide (NT-pro-BNP) are highly prevalent in coronary artery disease (CAD) patients with heart failure, and are associated with unfavorable prognosis. This study was undertaken to examine the relationship and prognostic impact of thyroid hormones, inflammatory biomarkers, and NT-pro-BNP on long-term outcomes in patients after acute coronary syndrome (ACS).

**Methods:**

The study comprised of 642 patients (age 58 ± 10 years, 77 % male) attending an in-patient cardiac rehabilitation program after experiencing ACS. Patients were evaluated for demographic, clinical and CAD risk factors as well as thyroid hormones (e.g., fT3, fT4 level, fT3/fT4 ratio), inflammatory biomarkers (hs-CRP, IL-6) and NT-pro-BNP levels. Data on fT3/fT4 ratio and NT-pro-BNP levels were not normally distributed and were natural-log transformed (ln). Both all-cause (cumulative) and cardiac-related mortality were considered the primary outcomes of interest.

**Results:**

According to the Cox model, age, NYHA class, (ln)NT-pro-BNP levels (HR 1.53, 95 % CI 1.13–2.07), fT4 level (HR 1.15, 95 % CI 1.04–1.27), and (ln)fT3/fT4 ratio (HR 0.08, 95 % CI 0.02–0.32) were the most important predictors of all-cause mortality among CAD patients after ACS. Similarly, age, NYHA class, (ln)NT-pro-BNP levels (HR 1.62, 95 % CI 1.11–2.36), fT4 (HR 1.15, 95 % CI 1.02–1.29) and (ln)fT3/fT4 ratio (HR 0.10, 95 % CI 0.02–0.55) independently predicted cardiac-related mortality. Kaplan-Meier analyses provided significant prognostic information with the highest risk for all-cause mortality in the low cut off measures of fT3/fT4 ratio <0.206 and NT-pro-BNP ≥290.4 ng/L (HR 2.03, 95 % CI 1.39–2.96) and fT4 level >12.54 pg/ml (HR = 2.34, 95 % CI 1.05–5.18). There was no association between hs-CRP, IL-6 and mortality in CAD patients after ACS.

**Conclusions:**

Thyroid hormones (i.e., fT4 level and fT3/fT4 ratio) together with NT-pro-BNP level may be valuable and simple predictors of long-term outcomes of CAD patients after experiencing ACS.

## Background

Thyroid hormones play a key role in cardiovascular regulation through various physiological and pathological manifestations [1, 2]. In recent years, increased attention has been focused on various circulating biologically active substances, collectively known as plasma biomarkers, and their utility in coronary artery disease (CAD) and heart failure (HF) prognosis [1–4]. Neurohormonal activation of N-terminal pro-B-type natriuretic peptides (NT-pro-BNP) remains one of the established markers for the detection and evaluation of HF severity and is considered a prognostic determinant of disease progression in CAD patients [5–7]. NT-pro-BNP is an important biomarker in patients with acute coronary syndrome (ACS) as it is a marker of myocardial cell necrosis and a strong predictor of morbidity and mortality [7, 8]. Increased NT-pro-BNP levels predict cardiac-related mortality in HF patients [9–11].

Altered thyroid function in the absence of primary thyroid disease is characterized by low total triiodothyronine (T3), free T3 (fT3) levels with normal total thyroxine (T4) and thyroid-stimulating hormone (TSH). This is due to reduced enzyme 5′ monodeiodinase activity, responsible for converting T4 into T3 in the peripheral tissues [12, 13]. It has been established that low circulating T3 levels are common in patients with HF [2, 13, 14], acute myocardial infarction (MI), [3, 15–17] and are linked with poor prognosis [18, 19]. The literature in this area indicates disparate perspectives on the association of thyroid hormones and NT-pro-BNP serum levels; some authors have demonstrated that higher thyroid function is associated with the higher NT-pro-BNP levels. In contrast others have suggested that hypothyroidism alters NT-pro-BNP concentrations [20, 21].

In CAD, the rupturing of a patient’s coronary plaque triggers an inflammatory cascade [22]. Studies have reported that interleukin-6 (IL-6) can stimulate BNP expression and that IL-6 has a significant positive association with NT-pro-BNP [23–25]. It has recently been hypothesized that IL-6, a pleiotropic proinflammatory cytokine, may exert an inhibitory effect on thyroid function and suggests the development of euthyroid sick syndrome in patients with systemic diseases [26–28].

Inflammatory marker C-reactive protein (CRP) is mainly produced in response to IL-6 and plays many pathophysiological roles in the inflammatory process in HF. It was shown that IL-6 and CRP are all inversely correlated with fT3 levels in patients with stable HF [29, 30].

Moreover, it has been demonstrated that the relative risk for CAD is higher in people with more inflammatory biomarkers such as CRP and IL-6 and NT-pro-BNP. These biomarkers also have the prognostic value of cardiovascular events in HF patients [22]. NT-pro-BNP, thyroid hormone and inflammatory biomarkers concentrations have also been found to change in patients treated with primary percutaneous coronary intervention following ACS [8, 13, 22]. However, the relationship between NT-pro-BNP, thyroid hormone and inflammatory biomarkers in patients undergoing a cardiac rehabilitation programme following ACS is not well described.

From a clinical perspective, understanding whether thyroid hormones and inflammatory biomarkers play a role in the relationship between NT-pro-BNP and mortality, would assist in greater accuracy with respect to the risk-stratification of CAD patients admitted to rehabilitation program and would also provide the opportunity for early interventions to address clinical risk factors and improve patient prognosis.

The primary objective of this study was to measure the associations between thyroid hormones, inflammatory biomarkers and NT-pro-BNP. The second aim of the study was to examine the potential impact of thyroid hormones, inflammatory biomarkers and NT-pro-BNP on long-term outcomes in patients enrolled in an in-patient cardiac rehabilitation program after ACS.

## Methods

### Study population

During the period from January 2006 until January 2013 a total of 826 CAD patients consecutively attending an inpatient cardiac rehabilitation program, at the Cardiovascular Rehabilitation Clinic of the Behavioral Medicine Institute of the Lithuanian University of Health Sciences in Palanga, Lithuania, were invited to participate in the study. All patients were admitted to the rehabilitation program after two weeks of in-hospital stationary treatment for ACS. Patients were excluded from the present study if they had history of thyroid or adrenal disease (*n* = 74), were actively taking thyroid medications or amiodarone (*n* = 34), had increased TSH (>3.7 μg/mL) concentrations indicating hypothyroidism (*n* = 14) or decreased TSH (<0.5 μg/mL) concentrations indicating hyperthyroidism (*n* = 10), had high concentrations of anti-thyroid peroxidase antibodies (anti-TPO ≥60 U/mL) indicating autoimmune thyroid disease (*n* = 15) or who declined the opportunity to participate in the study (*n* = 37). The final study population meeting inclusion/exclusion criteria was comprised of 642 CAD patients (77 % men; mean age, 58 ± 9 years) after ACS. All study participants took part in the exercise-based cardiac rehabilitation program and received standard treatment for secondary prevention of CAD according to the existing guidelines of secondary prevention through cardiac rehabilitation [31–33].

### Study design

This was a prospective study approved by The Regional Biomedical Research Ethics Committee. More details of study design, recruitment, and procedures have been published elsewhere [34, 35]. In short, the baseline measure was chosen to be patients’ assessment within three days of admission to the rehabilitation program, once they signed written informed consent. Eligible participants were evaluated for demographic factors (e.g., age and gender), clinical characteristics (e.g., New York Heart Association [NYHA] functional class, left ventricular ejection fraction [LVEF]), CAD risk factors (e.g., diabetes mellitus, smoking, hypertension, body mass index, and dyslipidemia) and current medication use. Blood samples were drawn for each participant to evaluate individual levels of thyroid hormones, NT-pro-BNP, high-sensitivity CRP (hs-CRP) and IL-6.

Follow-up data on mortality (time and cause of death) was used in the analysis as the primary outcome of interest. The data was primarily obtained from death certificates, post-mortem reports, and medical records. When data could not be obtained from these sources, the study team attempted to conduct telephone interviews with participant family members to obtain self-report mortality data or to contact the Causes of Death Register at the Institute of Hygiene of the Lithuanian Ministry of Health. In some cases, this outcome data was unavailable.

It should be noted that to be classified as a cardiac-related death, documentation of arrhythmias or cardiac arrest, death due to progressive HF or MI in the absence of a precipitating factor was required. Sudden unexpected death was classified as cardiac death when it occurred outside the hospital and was not followed up by autopsy. Death caused by accidents was excluded. Both cardiac-related and all-cause mortality (cumulative – death from any natural cause) were ascertained.

### Evaluation thyroid axis hormones, NT-pro-BNP, hs-CRP and IL-6 concentrations

All participants had a blood sample collection on second day after admission to the rehabilitation clinic. Venous blood samples were drawn after a minimum 12 h overnight fast for the evaluation of thyroid axis hormones, NT-pro-BNP, hs-CRP and IL-6 concentrations. Blood was centrifuged and serum was frozen at –40 °C. Serum concentrations of T3, fT3, T4, free T4 (fT4), TSH were analysed using automated enzyme immunoassay analyser AIA 600/21/1800 (Tosoh corporation, US) and radioimmunoassay kit RIA (R-EW-125, Belgium) for reverse T3 (rT3). Normal range for T3, from 70 to 170 ng/dL; fT3, from 2.0 to 4.0 pg/mL; rT3, from 9 to 35 nd/dl; T4, from 4,5 to 12.0 μg/dL; fT4, from 7.0 to 17.0 pg/mL; TSH, from 0.25 to 4.5 μIU/mL. Serum concentration of NT-pro-BNP and IL-6 were assessed using radio-immunoassay method (Roche Cobas analyser, Roche Diagnostics, UK). Normal serum concentration of NT-pro-BNP and IL-6 are <157 ng/L and from 0 to 7 pg/mL, respectively. Serum hs-CRP concentrations were assessed using the chemiluminescent immunoassay method (Beckman Coulter Unicel DXC 600) with normal values of ≤0.3 mg/dL.

### Statistical analysis

Data is expressed as mean ± standard deviation (SD) for variables with Gaussian distribution and as median (25th–75th percentile) for variables with non-normal distribution. Distribution of measurements was assessed using Kolmogorov-Smirnov test. Group comparisons were made using Student *t* test, Mann–Whitney *U* test, Fisher’s exact test, or chi-square test, as appropriate. Associations between continuous variables were assessed by Pearson product–moment analysis (Pearson r) or Spearman rank correlation analysis (Spearman r), as appropriate. Multivariate regression models were created for NT-pro-BNP, inflammatory biomarkers, and thyroid hormones adjusting for gender, age and body mass index. A two-tailed P value <0.05 was regarded as significant. All variables were examined for normal distribution and natural-log transformed (ln) when necessary. Univariate and multivariate Cox regression analyses were used to determine the relative risks (hazard ratio [HR]) for all-cause and cardiac-related mortality associated with demographic and clinical risk factors, such as age, gender, hypertension, NYHA class, and diabetes mellitus, as well as with NT-pro-BNP, thyroid hormones, thyroid hormones ratios, hs-CRP and IL-6 levels.

We also generated receiver operating characteristic (ROC) curves to assess cut-off values for demographic, clinical, thyroid hormones, inflammatory biomarkers and NT-pro-BNP characteristics that would prognostically discriminate between non-survivors (all-cause mortality) and survivors. We used the approach by DeLong et al. [36] to compare the areas under the ROC curves (AUCs) of demographic and clinical models with AUCs from biomarkers and additive models.

Then, based on the cut-off values patients outcomes were assessed using Kaplan-Meier curves; a log-rank (Mantel-Cox) test was used to compare survival curves and estimate HR. All tests were performed using software SPSS for Windows, version 17.0 (SPSS Inc., Chicago, USA) and MedCalc for Windows, version 12.5 (MedCalc Software, Ostend, Belgium).

## Results

Baseline socio-demographic variables, clinical characteristics, CAD risk factors and the mean concentrations of thyroid hormones, NT-pro-BNP, IL-6, hs-CRP and current treatment of 642 patients are shown in Table 1.

**Table 1:** Patients characteristics

Characteristics	All	Survived throughout follow-up	Died during follow-up	p-value
	*n* = 642	*n* = 607	*n* = 35	
Age (years), mean ± SD	57.6 ± 9.5	57.4 ± 9.4	62.3 ± 9.2	0.004
Gender, n (%)				0.382
Men	493 (76.8)	464 (76.4)	29 (82.9)	
Women	149 (23.2)	143 (23.6)	6 (17.1)	
Body mass index, mean ± SD	29.8 ± 4.7	29.9 ± 4.7	29.0 ± 5.2	0.355
Diagnosis, n (%)				0.010
Angina pectoris	159 (24.8)	149 (24.6)	10 (28.6)	
Acute myocardial infarction	405 (63.1)	381 (62.8)	24 (68.6)	
Previous myocardial infarction	78 (12.1)	77 (12.7)	1 (2.9)	
Angina pectoris class, n (%)				0.063
I	143 (22.3)	138 (22.8)	5 (14.3)	
II	214 (33.3)	196 (32.3)	18 (51.4)	
III	18 (2.8)	16 (2.6)	2 (5.7)	
NYHA, n (%)				<0.001
I	52 (8.1)	52 (8.6)	-	
II	468 (72.9)	451 (74.2)	17 (48.6)	
III	122 (19.0)	104 (17.2)	18 (51.4)	
Hypertension, n (%)				0.001
Grade 1 (mild)	39 (6.1)	37 (6.1)	2 (5.7)	
Grade 2 (moderate)	354 (55.2)	345 (56.8)	9 (25.7)	
Grade 3 (severe)	109 (17.0)	96 (15.8)	13 (37.1)	
Left ventricular ejection fraction %, mean ± SD	50.2 ± 9.0	50.5 ± 8.9	43.4 ± 8.2	0.001
Diabetes mellitus, n (%)	74 (11.5)	67 (11.1)	7 (20.0)	0.107
Nitrate, n (%)	255 (39.7)	233 (38.4)	22 (62.9)	0.004
Beta-blockers, n (%)	557 (86.8)	529 (87.3)	28 (80.0)	0.214
Angiotensin-converting enzyme inhibitors, n (%)	527 (82.1)	496 (81.8)	31 (88.6)	0.312
Diuretic, n (%)	101 (15.7)	92 (15.2)	9 (25.7)	0.096
N-terminal pro-B-Type natriuretic peptide (ng/L), median (interquartile ranges)	328.3 (120.8–770.5)	303.7 (115.7–709.7)	570.0 (305.5–299.0)	0.001
hs-C-reactive protein (mg/dL), median (interquartile ranges)	0.26 (0.12–0.58)	0.26 (0.12–0.57)	0.47 (0.13–0.88)	0.077
Interleukin-6 (pg/ml), median (interquartile ranges)	3.1 (1.7–5.5)	3.1 (1.7–5.5)	3.7 (2.1–8.0)	0.209
Reverse Triiodothyronine (ng/ml), median (interquartile ranges)	0.27 (0.22–0.37)	0.27 (0.22–0.36)	0.30 (0.20–0.43)	0.375
Free Triiodothyronine (pg/ml), median (interquartile ranges)	2.80 (2.55–3.06)	2.80 (2.56–3.06)	2.72 (2.24–3.00)	0.036
Thyroid-stimulating hormone (μIU/ml), median (interquartile ranges)	1.8 (1.2–2.6)	1.8 (1.2–2.8)	1.7 (1.4–2.5)	0.875
Free Thyroxine (pg/ml), mean ± SD	13.0 ± 2.5	12.9 ± 2.4	14.6 ± 3.9	<0.001
Total Triiodothyronine (ng/dl), mean ± SD	106.7 ± 19.0	106.8 ± 19.0	104.3 ± 18.3	0.519
Total Thyroxine (μg/dl), mean ± SD	7.1 ± 1.5	7.8 ± 1.5	8.4 ± 1.4	0.042
Follow-up (months), mean ± SD	52.0 ± 29.2	52.5 ± 29.1	43.7 ± 30.6	0.029

*NYHA* New York Heart Association functional class

During the follow-up period (i.e., maximum of 118 months) there were 23 cardiac-related and 35 all-cause deaths. Death most frequently occurred following MI and the patients who died tended to be older, have a higher NYHA functional class, lower LVEF, have more severe hypertension, were more likely to have used nitrates and to have higher concentrations of NT-pro-BNP, hs-CRP, T4, fT4 and lower concentrations of fT3 during the rehabilitation period at baseline assessment, as compared to survivors (Table 1).

### Associations between thyroid hormones, hs-CRP, IL-6 and NT-pro-BNP

There were significant correlations between (ln)fT3 and fT4 levels (*r* = 0.179, *p* < 0.001), and (ln)fT4 and T3 (*r* = 0.112, *p* = 0.02). Significant correlations were also found between (ln)rT3 and fT4 (*r* = 0.776, *p* < 0.001). There were also strong correlation between (ln)hs-CRP and (ln)IL-6 serum concentrations (*r* = 0.650, *p* < 0.001) and between (ln)IL-6 and fT4 (*r* = 0.091, *p* = 0.03). The univariate regression analysis indicated that higher (ln)NT-pro-BNP levels were negatively correlated with concentrations of (ln)fT3 (β = −0.112, *p* = 0.005), positively correlated with concentrations of T4 (β = 0.126, *p* = 0.009), and (ln)TSH (β = 0.097, *p* = 0.015). Biomarker (ln)NT-pro-BNP correlated positively with fT4 (β = 0.100, *p* = 0.012). A lower (ln)fT3/fT4 ratio was associated with higher (ln)NT-pro-BNP (β = −0.112, *p* = 0.004). The positive association between (ln)hs-CRP and (ln)NT-pro-BNP (β = 0.146, *p* < 0.001), (ln)IL-6 (β = 0.228, *p* < 0.001, respectively) was determined.

However, multivariate linear regression models, adjusted for age, gender and body mass index, revealed that (ln)NT-pro-BNP remain associated with hs-CRP (β = 0.59, *p* < 0.001), (ln)IL6 (β = 0.254, *p* < 0.001), fT4 (β = 0.100, *p* = 0.011), and T4 (β = 0.112, *p* = 0.019).

### Prognostic impact of thyroid hormones and NT-pro-BNP

Univariate analysis of data showed that age, NYHA class, (ln)NT-pro-BNP, (ln)rT3, fT4, (ln)fT3, and (ln)fT3/fT4 ratio were associated with all-cause mortality. Moreover age, NYHA class, (ln)NT-pro-BNP, (ln)rT3, fT4, and (ln)fT3/fT4 ratio were associated with cardiac-related mortality (Table 2). A multivariate Cox regression model showed that age (HR 1.06, 95 % CI 1.02–1.11, *p* = 0.003), NYHA class (HR 3.01, 95 % CI 1.44–6.23, *p* = 0.003), (ln)NT-pro-BNP levels (HR 1.53, 95 % CI 1.13–2.07, *p* = 0.006), fT4 level (HR 1.15, 95 % CI 1.04–1.27, *p* = 0.005), and (ln)fT3/fT4 ratio (HR 0.08, 95 % CI 0.02–0.32, *p* < 0.001) were associated with all-cause mortality. However, NYHA class (HR 3.58, 95 % CI 1.43–8.94, *p* = 0.006), (ln)NT-pro-BNP levels (HR 1.62, 95 % CI 1.11–2.36, *p* = 0.013), fT4 level (HR 1.15, 95 % CI 1.02–1.29, *p* = 0.018) and (ln)fT3/fT4 ratio (HR 0.10, 95 % CI 0.02–0.55, *p* = 0.008) remained independent predictors of cardiac-related mortality. The hs-CRP and IL-6 levels were not found to be associated with mortality (Table 2).

**Table 2:** Cox regression analyses for factors associated with all-cause and cardiac-related mortality

Variable	Univariate	p-value	Multivariate^a^	p-value
	HR (95 % CI)		HR (95 % CI)	
All-cause mortality				
Age	1.07 (1.03–1.11)	0.001	1.06 (1.02–1.11)	0.003
Gender	0.67 (0.28–1.61)	0.368	0.52 (0.21–1.27)	0.150
Arterial Hypertension	0.98 (0.79–1.21)	0.821	0.97 (0.78–1.20)	0.785
Diabetes mellitus	2.26 (0.98–5.20)	0.055	2.00 (0.85–4.68)	0.111
NYHA class	4.32 (2.23–8.35	<0.001	3.01 (1.44–6.29)	0.003
(ln)NT-pro-BNP	1.56 (1.16–2.11)	0.004	1.53 (1.13–2.07)	0.006
(ln)hs-CRP	1.32 (0.99–1.76)	0.062	1.26 (0.93–1.71)	0.137
(ln)IL-6	1.39 (0.90–2.14)	0.138	1.21 (0.77–1.90)	0.398
fT4	1.17 (1.06–1.28)	0.002	1.15 (1.04–1.27)	0.005
(ln)fT3	0.12 (0.02–0.96)	0.045	0.14 (0.01–1.36)	0.089
(ln)fT3/fT4	0.09 (0.03–0.31)	<0.001	0.08 (0.02–0.32)	<0.001
(ln)rT3	2.29 (1.11–4.73)	0.025	2.15 (1.01–4.58)	0.049
Cardiac-related mortality				
Age	1.06 (1.01–1.11)	0.023	1.05 (1.00–1.10)	0.062
Gender	1.12 (0.44–2.84)	0.815	0.79 (0.30–2.06)	0.623
Arterial Hypertension	1.02 (0.78–1.34)	0.871	1.06 (0.81–1.38)	0.688
Diabetes mellitus	1.35 (0.40–4.56)	0.627	1.08 (0.31–3.76)	0.899
NYHA class	6.01 (3.57–14.05)	<0.001	3.58 (1.43–8.94)	0.006
(ln)NT-pro-BNP	1.65 (1.13–2.41)	0.009	1.62 (1.11–2.36)	0.013
(ln)hs-CRP	1.12 (0.78–1.60)	0.537	1.08 (0.75–1.56)	0.667
(ln)IL-6	1.41 (0.82–2.40)	0.213	1.35 (0.77–2.34)	0.293
fT4	1.17 (1.04–1.31)	0.007	1.15 (1.02–1.29)	0.018
(ln)fT3	0.13 (0.01–1.71)	0.122	0.23 (0.02–3.49)	0.287
(ln)fT3/fT4	0.09 (0.02–0.43)	0.002	0.10 (0.02–0.55)	0.008
(ln)rT3	2.46 (1.02–5.29)	0.045	2.05 (0.81–5.16)	0.128

*HR* hazard ratio, *CI* confidence interval, *ln* natural logarithm, *hs-CRP* high-sensitivity C-reactive protein, *fT3* Free Triiodothyronine, *fT4* Free Thyroxine, *IL-6* Interleukin-6, *NT-pro-BNP* N-terminal pro-B-Type natriuretic peptide, *rT3* Reverse triiodothyronine, *NYHA* New York Heart Association functional class

^a^Final model: adjusted for age, arterial hypertension, diabetes mellitus, peripheral arterial disease

**Fig. 1:**
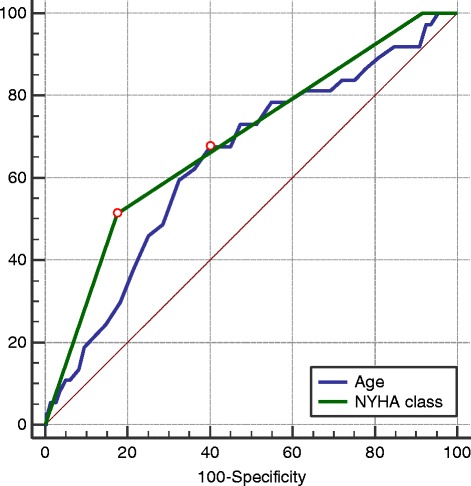
Receiver operating characteristic curves for age and NYHA class predicting all-cause mortality during follow up. NYHA – New York Heart Association functional class. Area under the curve of age was 0.642 (95 % confidence interval [CI], 0.604–0.678; *p* = 0.003), of NYHA 0.690 (95 % CI, 0.653–0.725; *p* < 0.001)

**Fig. 2:**
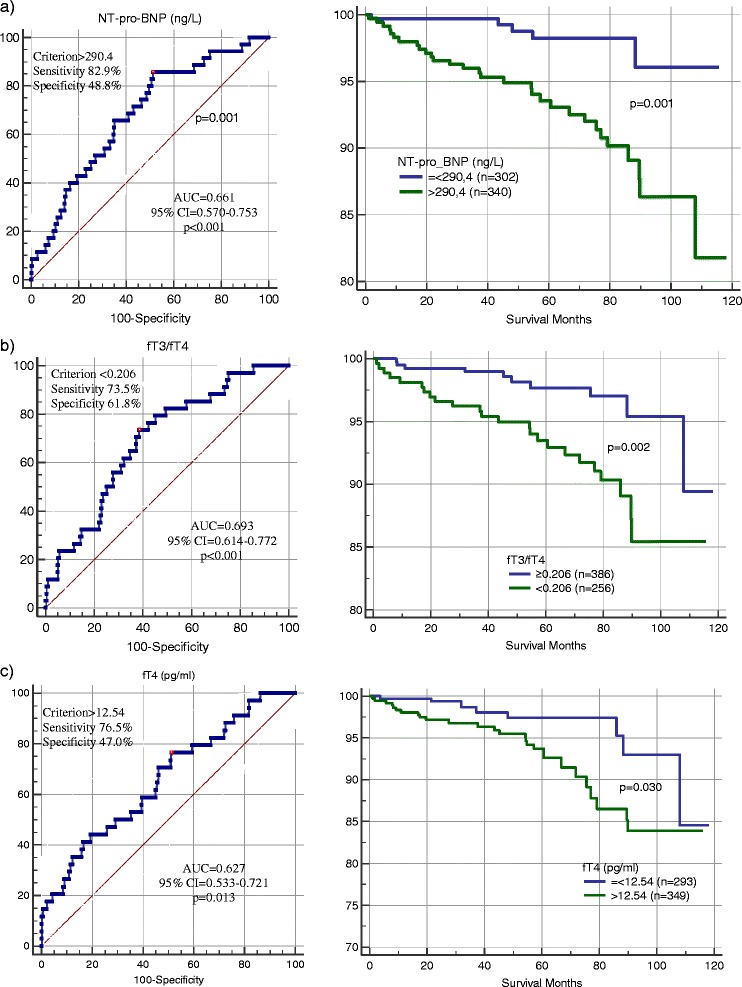
Receiver operating characteristics curves and Kaplan-Meier survival plots for **a** NT-pro-BNP, **b** fT3/fT4 ratio, and **c** fT4 values for predicting all-cause mortality during follow-up. Results are the area under the curve (AUC) and associated 95 % confidence interval (CI). The best cut-off values are labeled. A log-rank test was used to compare survival curves. NT-pro-BNP – N-terminal pro-B-Type natriuretic peptide, fT3/fT4 – Free Triiodothyronine/Free Thyroxine ratio, fT4 – Free Thyroxine

**Fig. 3:**
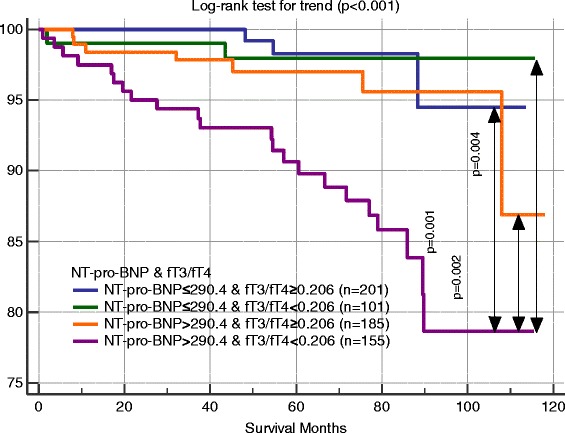
Kaplan-Meier survival plots for all-cause mortality for 642 CAD patients stratified into 4 groups based on cut-off values for NT-proBNP (290.4 ng/L) and ratio fT3/fT4 (0.206) levels. fT3/fT4 – Free Triiodothyronine/Free Thyroxine ratio; NT-pro-BNP – N-terminal pro-B-Type natriuretic peptide. The group with low fT3/fT4 ratio (<0.206) and high NT-proBNP (>290.4 ng/L) (*bottom line*) had a worse prognosis than others groups (*p* < 0.001; by the log-rank test)

Figure 1 shows ROC curves for age and NYHA class. Figures 2 and 3 show ROC curves and Kaplan-Meier survival plots for biomarkers characteristics predicting all-cause mortality. When participants were stratified into high and low NT-pro-BNP level groups (cut-off 290.4 ng/L), the outcome (cumulative survival) for patients with high NT-pro-BNP levels was significantly different in comparison to patients with low NT-pro-BNP levels (HR 3.79, 95 % CI 1.57–9.15, *p* = 0.003) (Fig. 2a). When patients were stratified into high and low fT3/fT4 ratio groups (cut-off of <0.206), the outcome for patients with lower fT3/fT4 ratio was significantly worse than for patients with a higher fT3/fT4 ratio (HR 2.99, 95 % CI 1.43–6.26, *p* = 0.004) (Fig. 2b). ROC analysis was used to assess the best cut-off values for fT4 levels (cut-off 12.54 pg/ml) predicting all-cause mortality (AUC 0.63, 95 % CI 0.53–0.72, *p* = 0.013). When we stratified participants into two groups according to cut-off values, we found that higher fT4 levels were associated with worse outcomes (HR 2.34, 95 % CI 1.05–5.18, *p* = 0.036) (Fig. 2c). When patients were subdivided based on the cut-off values for fT3/fT4 ratio (of <0.206) and NT-pro-BNP (≥290.4 ng/L) levels, the outcomes for patients with a lower fT3/fT4 ratio and higher NT-pro-BNP were worse, as compared with the other groups (HR 2.03, 95 % CI 1.39–2.96, *p* < 0.001) (Fig. 3).

**Fig. 4:**
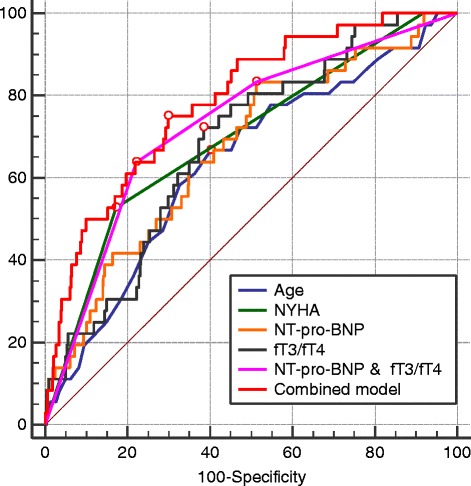
Receiver operating characteristics curves of different parameters and models for predicting all-cause mortality during follow-up. NT-pro-BNP – N-terminal pro-B-Type natriuretic peptide, fT3/fT4 – Free Triiodothyronine/Free Thyroxine ratio, NYHA – New York Heart Association functional class. Combined model - composed by parameters of age, NYHA class, NT-pro-BNP, and fT3/fT4

Data on how the AUCs from the demographic and clinical predictors compares with the biomarkers alone or combined models are presented in Table 3 and Fig. 4. Overall, age and NYHA class showed good discriminatory power for predicting all-cause mortality during follow up. The NT-pro-BNP level and fT3/fT4 ratio model had the best AUC of all isolated parameters. A combined model comprising of age, NYHA class, NT-pro-BNP level, and fT3/fT4 ratio showed the highest discriminatory power among other predictors.

**Table 3:** Receiver operating characteristic (ROC) curve evaluation for all-cause mortality prediction

Prediction of all-cause mortality: ROC analysis	Area under the curve (AUC)	95 % CI	p
Age	0.642	0.604–0.678	0.003
NYHA class	0.690	0.653–0.725	<0.001
NT-pro- BNP	0.675	0.638–0.711	<0.001
fT3/fT4	0.684	0.647–0.719	<0.001
NT-pro-BNP & fT3/fT4 model	0.732	0.693–0.766	<0.001
Combined model	0.800	0.753–0.817	<0.001
ROC curves comparison – Combined model vs:	AUC (difference)	95 % CI	p
Age	0.150	0.064–0.235	<0.001
NYHA class	0.090	0.030–0.150	0.003
NT-pro-BNP	0.115	0.023–0.207	0.014
fT3/fT4	0.103	0.034–0.173	0.004
NT-pro-BNP & fT3/fT4 model	0.055	−0.032–0.142	0.218

*CI* confidence interval, *NT-pro-BNP* N-terminal pro-B-Type natriuretic peptide, *fT3/fT4* Free Triiodothyronine/Free Thyroxine ratio, *fT4* Free Thyroxine, *NYHA* New York Heart Association functional class; Combined model - composed by parameters of age, NYHA, NT-pro-BNP, and fT3/fT4

Evaluating all-cause mortality, the model comprised of age, NYHA class, NT-pro-BNP level, and fT3/fT4 ratio was significantly better than age (difference between AUC: 0.150, 95 % CI 0.064–0.235, *p* < 0.001), NYHA class (difference between AUC: 0.090, 95 % CI 0.030–0.150, *p* = 0.003), NT-pro-BNP level (difference between AUC: 0.115, 95 % CI 0.023–0.207, *p* = 0.014), and fT3/fT4 ratio (difference between AUC: 0.103, 95 % CI 0.034–0.173, *p* = 0.218). However, it failed to perform significantly better than NT-pro-BNP level and fT3/fT4 ratio model.

## Discussion

Our study demonstrated a significant relationship between the suppression of thyroid axis function, increased inflammation markers and increased NT-pro-BNP levels in CAD patients undergoing rehabilitation after ACS. We have also demonstrated that measures of fT4 and fT3/fT4 ratio together with NT-pro-BNP can be important prognostic markers of negative long-term outcomes (i.e., mortality). We found no association between hs-CRP and IL-6 and mortality among CAD patients.

A positive relationship between hs-CRP, IL-6 and NT-pro-BNP was established in our study indicating that thyroid function disturbances contribute to inflammatory processes even during the rehabilitation period (i.e., after acute coronary events). Numerous studies have confirmed fT3 and low-T3 syndrome as predictors of elevated NT-pro-BNP [7, 37]. The correlation of fT3 and NT-pro-BNP was weaker in our study compared to Pfister et al. [37], which might be due to the dominant influence of cardiac abnormalities on NT-pro-BNP values in our patients’ population. However, association of fT4 and NT-pro-BNP was evident in our study. Mayer et al. [38] showed that even mild changes of fT4, still within normal clinically accepted levels, may influence the burden of HF as quantified by its biological surrogates (i.e., natriuretic peptides). Free thyroxine concentrations were significantly positively associated with NT-pro-BNP and BNP in a continuous manner. In this study association was more evident at high-normal fT4 levels, than at low-normal, where only the increase of a more sensitive marker (NT-pro-BNP) was observed. Our findings add to the developing understanding of the relationship between NT-pro-BNP, thyroid hormones and inflammatory biomarkers in CAD patients during rehabilitation after ACS. Specifically, that NT-pro-BNP and thyroid hormone level during rehabilitation could be an important predictor of negative outcomes. Determining whether these particular associations remain the same after rehabilitation, and whether changes in these associations are related to long term outcomes of CAD patients is beyond the scope of this study but should be subject of further investigation.

In the numerous prognostic studies, the altered thyroid metabolism assessed by various parameters, indicated disturbed T4 peripheral conversion, such as a reduction in circulating levels of T3 [18, 39, 40], low T3 syndrome [19, 41–44], mild changes of fT4 [45], a high rT3 level [46], a low fT3/rT3 ratio [46] and a low fT3/fT4 ratio in serum [45].

Passino et al. [20] showed that monitoring the combination of low fT3 and high BNP levels is a useful approach for determining long-term prognosis in patients with advanced HF. Irrespective of the parameters used, all these studies showed that altered peripheral thyroid hormones pathways were associated with a high incidence of fatal events (cumulative or cardiac-related death). Low fT3 levels are the most common alternation of thyroid hormones metabolism in patients with HF and are related with the severity of HF. Presently, low fT3 levels are found in approximately 20–30 % of patients with overt HF, 58 % of patients with advanced HF and in less than 10 % of those with early HF [47]. Low T3 reduction is more frequent in patients with NYHA classes III-IV [12]. In the present study with CAD patients group (73 % with a NYHA II functional class, 63 % after acute MI) the fT3 levels have not exposed prognostic impact to the long-term mortality. This might be due to dominant influence of cardiac abnormalities on NT-pro-BNP values in our cohort or to the recovered T3 level after ACS during the rehabilitation period [17]. Friberg et al. [17] showed that patients with poor heart function or who experienced a more intense inflammatory reaction to acute MI, showed more pronounced down-regulation of the thyroid hormones. The mean levels of the T3 declined between the first 6 h and the 24–36 h period after acute MI, although T3 levels were found to recover within 12 weeks [17].

Our study also revealed that age, NYHA class, (ln)NT-pro-BNP, fT4 and (ln)fT3/fT4 ratio were independent predictors of all-cause mortality and that NYHA class, (ln)NT-pro-BNP, fT4, (ln)fT3/fT4 ratio remained independent predictors of cardiac-related mortality. Additionally, it was demonstrated that higher levels of NT-pro-BNP (≥290.4 ng/L) and a lower fT3/fT4 ratio (<0.206) were independent predictors of cumulative mortality. When patients were subdivided based on the cut-off values for fT3/fT4 ratio and NT-pro-BNP levels, the outcome for the patient group with the lower fT3/fT4 ratio and higher NT-pro-BNP were found to be worse, as compared with the other 3 groups.

However, there was no association between inflammatory markers and mortality in our study. Conversely, some authors have suggested that hs-CRP > 3.90 mg/L and NT pro-BNP > 2247 pmol/L are associated with all-cause mortality in dilated cardiomyopathy patients [48, 49] and can be a potential predictors for all-cause mortality [50].

Euthyroid sick syndrome is the most common type of thyroid hormones abnormality where elevation of fT4 levels is frequently found. Friberg et al. [17, 40] showed that rT3, the inactive metabolite of T3, is positively correlated with its precursor fT4, which suggests that fT4 levels can also be used instead of rT3. Higher levels of rT3 and fT4 were associated with worse rates of survival, that is, patients that died within the first week after acute MI had a median free fT4 level of 16 pmol/L as compared with 15 pmol/L in survivors. Similar differences were also seen after 1 month and 1 year. It was also noted that in patients with a history of angina pectoris, the highest levels of fT4 were found in patients classified with unstable angina pectoris. Jung et al. [51] examined the association of thyroid function with CAD in euthyroid angina pectoris patients and showed that fT4 levels were associated with the presence and the severity of CAD. Jung et al. [51] study also suggests that elevated serum fT4 levels, even within normal range, could be a risk factor for CAD. Our study extends the data of Jung et al. [51] by showing that elevated fT4 levels during rehabilitation are related with long-term mortality in CAD patients.

Kozdag et al. [45] studied a group of 111 patients with ischemic and non-ischemic dilated cardiomyopathy and advanced NYHA class (III-IV), and showed that patients with lower fT3/fT4 ratio had a significantly less favorable outcome, as compared with those having higher fT3/fT4 ratio during 12 months of follow-up. Studies also find that NT-pro-BNP levels predict cardiac-related mortality in HF patients and patients with congenital heart disease [9–11, 52]. Recent review suggests that NT-pro-BNP assessment alone could be a useful tool predicting all-cause mortality in stable CAD patients [53]. However, we found that not only higher levels of NT-pro-BNP but also fT4 and lower fT3/fT4 ratio during cardiac rehabilitation were associated with long-term mortality in patients after ACS. Thus, in practical terms, the determination of fT4 level and fT3/fT4 ratio together with NT-pro-BNP, are valuable and simple predictors for the identification of CAD patients that are more likely to experience negative long-term outcomes after cardiac rehabilitation.

However, despite this relationship, using fT4 levels as a prognostic indicator in clinical settings will require more research to establish its validity. For example, future research could look at the influence of NT-pro-BNP and inflammatory markers concentration changes on fT4 levels in order to establish possible mediation/moderation effects on mortality. In the present form our findings may not be clinically relevant, but should be used to create multifactorial risk for mortality models.

Despite our consistent results, there were some limitations of the study that should be noted. Particularly, in terms of generalizability, as the majority of study participants had mild to moderate HF and all study patients attended a single-center in-patient cardiac rehabilitation program after being treated for ACS. Thus, the results may not apply to patients with more advanced HF and those who do not attend cardiac rehabilitation programs. Another limitation of our study was that we did not measure the fT3 level and other thyroid hormones in acute stage of coronary syndromes and therefore did not established variations of the fT3 before cardiac rehabilitation.

## Conclusions

This study demonstrated that thyroid hormones characteristics such as fT4 level and fT3/fT4 ratio together with NT-pro-BNP level may be valuable predictors of long-term outcomes of CAD patients after experiencing ACS. Additional research is needed to determine how and when these prognostic indicators should be operationalized in a clinical setting and how treatment should be prioritized in higher-risk cardiac rehabilitation patients.

## Abbreviations

ACS: acute coronary syndrome
CAD: coronary artery disease
CRP: C-reactive protein
fT3: free T3
fT4: free T4
HF: heart failure
HR: hazard ratio
hs-CRP: high-sensitivity CRP
IL-6: interleukin-6
ln: natural-log transformed
LVEF: left ventricular ejection fraction
MI: myocardial infarction
NT-pro-BNP: N-terminal pro-B-type natriuretic peptides
NYHA: New York Heart Association
rT3: reverse T3
SD: standard deviation
T3: total triiodothyronine
T4: total thyroxine
TSH: thyroid-stimulating hormone
